# Emotional Responses to Suicidal Patients: Factor Structure, Construct, and Predictive Validity of the Therapist Response Questionnaire-Suicide Form

**DOI:** 10.3389/fpsyt.2018.00104

**Published:** 2018-04-05

**Authors:** Shira Barzilay, Zimri S. Yaseen, Mariah Hawes, Bernard Gorman, Rachel Altman, Adriana Foster, Alan Apter, Paul Rosenfield, Igor Galynker

**Affiliations:** ^1^Department of Psychiatry, Icahn School of Medicine at Mount Sinai, New York City, NY, United States; ^2^Department of Psychiatry and Behavioral Health, Mount Sinai Beth Israel Medical Center, New York City, NY, United States; ^3^Gordon F. Derner School of Psychology, Adelphi University, Garden City, NY, United States; ^4^Herbert Wertheim College of Medicine, Florida International University, Miami, FL, United States; ^5^Feinberg Child Study Center, Schneider Children’s Medical Center, Petach Tikva and Sackler Faculty of Medicine, Tel Aviv University, Tel Aviv, Israel; ^6^Department of Psychiatry, Mount Sinai St. Luke’s, New York City, NY, United States

**Keywords:** suicide, countertransference, emotional response, risk assessment, suicidal ideation, suicide attempt, suicide prevention, TRQ

## Abstract

**Background:**

Mental health professionals have a pivotal role in suicide prevention. However, they also often have intense emotional responses, or countertransference, during encounters with suicidal patients. Previous studies of the Therapist Response Questionnaire-Suicide Form (TRQ-SF), a brief novel measure aimed at probing a distinct set of suicide-related emotional responses to patients found it to be predictive of near-term suicidal behavior among high suicide-risk inpatients. The purpose of this study was to validate the TRQ-SF in a general outpatient clinic setting.

**Methods:**

Adult psychiatric outpatients (*N* = 346) and their treating mental health professionals (*N* = 48) completed self-report assessments following their first clinic meeting. Clinician measures included the TRQ-SF, general emotional states and traits, therapeutic alliance, and assessment of patient suicide risk. Patient suicidal outcomes and symptom severity were assessed at intake and one-month follow-up. Following confirmatory factor analysis of the TRQ-SF, factor scores were examined for relationships with clinician and patient measures and suicidal outcomes.

**Results:**

Factor analysis of the TRQ-SF confirmed three dimensions: (1) affiliation, (2) distress, and (3) hope. The three factors also loaded onto a single general factor of negative emotional response toward the patient that demonstrated good internal reliability. The TRQ-SF scores were associated with measures of clinician state anger and anxiety and therapeutic alliance, independently of clinician personality traits after controlling for the state- and patient-specific measures. The total score and three subscales were associated in both concurrent and predictive ways with patient suicidal outcomes, depression severity, and clinicians’ judgment of patient suicide risk, but not with global symptom severity, thus indicating specifically suicide-related responses.

**Conclusion:**

The TRQ-SF is a brief and reliable measure with a 3-factor structure. It demonstrates construct validity for assessing distinct suicide-related countertransference to psychiatric outpatients. Mental health professionals’ emotional responses to their patients are concurrently indicative and prospectively predictive of suicidal thoughts and behaviors. Thus, the TRQ-SF is a useful tool for the study of countertransference in the treatment of suicidal patients and may help clinicians make diagnostic and therapeutic use of their own responses to improve assessment and intervention for individual suicidal patients.

## Introduction

Clinicians’ emotional responses to their patients, broadly referred to as *countertransference*, have important implications for treatment outcomes (1, 2). Countertransference, which emerges in the context of a therapeutic relationship with the patient, has been extensively addressed in the theoretical and qualitative clinical literature (2–4). The concept of countertransference has developed over the years since Freud (1910) classical definition of countertransference as the therapist’s own unresolved conflict-based reactions to the patient’s transference, which are unbeneficial to treatment (5) to a “total” view of countertransference as comprising all of a clinician’s emotional responses to a patient (6), an important source of information for understanding the patient’s dynamics. Following the growing body of quantitative empirical research on countertransference over the past three decades, several recent studies have provided quantitative empirical evidence supporting the relation of countertransference to treatment outcomes (2, 7–9). Clinicians’ feelings toward their patients may relate to treatment outcomes both as a causal force, influencing clinician behavior (3) and the therapeutic bond (4), and as a diagnostic signal, detecting patient patterns indicative of difficulties that will persist into the future (3, 10).

Assessing and treating patients at risk for suicide are clinical domains for which the study of countertransference is particularly pertinent, given the highly stressful nature of the interactions (11–14) and the concomitantly powerful feelings suicidal patients elicit, such as fear, anxiety, frustration, incompetence, helplessness, discouragement, sadness, and anger (15–18). The experience of bearing a patient’s intense despair and hopelessness is extraordinarily difficult, and can erode clinicians’ own sense of hope. For example, Pompili et al. (19) found that affective temperaments were significantly associated with hopelessness among patients with bipolar disorders, and hopelessness was associated with at least threefold odds for suicidal risk.

Suicide is one of the leading causes of death in the US and around the world (20), and much opportunity for prevention may be missed, as an estimated one-third of individuals (21) who die by suicide encounter a mental health-care provider within 1 month of their death (21–24). Thus, there is both a need for better understanding of the interactions between clinicians and suicidal patients, and for scalable and psychometrically sound ways of examining such interactions in large studies as well as clinical settings. Further, if areas of improvement in clinicians’ management of emotional responses to patients can be identified and tracked, this will facilitate training and improve suicide outcomes.

To this end, we developed a brief questionnaire to specifically target countertransference that is potentially indicative of a patient’s short-term suicide risk—the Therapist Response Questionnaire-Suicide Form (TRQ-SF). The questionnaire, described in detail in our previous study (25) comprises five items derived from the 79-item Therapist Response Questionnaire (TRQ; originally called the Countertransference Questionnaire (3, 26);) two items from the therapist form of the Working Alliance Inventory [WAI (27, 28)], and three rational items developed *de novo* by our group to capture distinctive emotional responses to high-risk suicidal patients (25). In our pilot study of this measure, the TRQ-SF was administered to first-year psychiatry residents treating patients psychiatrically hospitalized in the context of acute suicidal thoughts and behaviors [STB (25)]. In that study, although a two-factor model comprising dimensions of (a) Affiliation (vs. Rejection)—characterized by five items, and (b) Distress (vs. Comfort)—characterized by three items, fit the data, we also included a third countertransference dimension that was clinically and conceptually coherent: Hopefulness (vs. Hopelessness)—comprising two significantly correlated items with strong face validity that did not load robustly on either of the preceding factors. We found that level of patient STB in the 1–2 months following hospital discharge was significantly correlated with clinician Distress (*r* = 0.27, *p* = 0.02) and Hopefulness (*r* = −0.25, *p* = 0.03), and more robustly correlated with the interaction of Distress and Hopefulness (*r* = 0.42, *p* < 0.001). In addition, a composite total score for the TRQ-SF was found to predict post-discharge suicidal behavior among high-risk psychiatric inpatients (29), and clinicians’ judgment of suicide risk in psychiatric outpatients (9).

While the initial results were promising, further research is needed to generalize these findings to other mental health professionals, patient populations, and treatment settings. Moreover, the previous study was limited by small sample size, and fully anonymous collection of clinician data that made accounting for individual clinician differences impossible. Therefore, in the present study, we seek to validate the TRQ-SF as a measure of suicide-risk associated countertransference in a general outpatient sample treated by a larger sample of clinicians varying in professional degree, experience and orientation. Specifically we aim to (a) confirm the previously proposed three-factor structure (b) provide further support for the reliability and validity of the scale and (c) evaluate the performance of the TRQ-SF in a different treatment setting with a larger, more diverse clinician and patient sample. We explore the following hypotheses:

1. factor Structure and Reliability:
  (a) the three-factor structure of the TRQ-SF proposed by Yaseen et al. (25) will be replicated in the current sample.
  (b) The TRQ-SF and its subscales will demonstrate good reliability evidenced by high Cronbach’s alphas.
2. convergent and discriminant validity:
  The TRQ-SF will be associated with measures of clinician general emotional state and therapeutic alliance, while being unrelated to clinician personality traits.
3. concurrent and prospective criterion validity:
  The TRQ will demonstrate both cross-sectional and predictive relationships with patient suicide outcome measures, as well as robust associations with clinicians’ concurrent judgment of patient suicide risk.

## Materials and Methods

### Participants and Procedures

We recruited 346 patient and 48 clinician participants from adult psychiatric outpatient centers serving urban populations in New York City between January 2016 and June 2017. Patients presenting for intake or first visit with a new provider for any pharmacological or psychosocial treatment at one of three outpatient centers were referred by the evaluating clinician to an ongoing suicide risk study (MARIS) (29). Any mental health professional conducting patient intakes at one of the participating outpatient clinics was eligible to participate in the study. Eligible clinicians were approached at the onset of the study or of their employment at the clinic. Clinicians provided informed consent and completed a baseline packet of questionnaires assessing demographic and trait characteristics. Clinicians were instructed to identify patients who were potentially eligible to participate in the study at their first meeting with the patient. Clinicians then completed a packet including questionnaires about their clinical assessments, emotional responses, and emotional state when meeting with that particular patient. Of 81 potential clinicians, 64 (79%) consented to participate and 48 (59%) had at least one patient participant enrolled in the study.

Eligible patients who indicated to their clinician that they were interested in participating were contacted by an affiliate of the study and completed a baseline assessment following informed consent. Patients were eligible to participate in the study if they were over 18 years old and were meeting the referring clinician for the first time. Patients were excluded from the study if follow-up would be impaired—they were homeless or lacked collateral means of contact, were unable to understand the consent or did not speak English, or suffered a physical or mental impairment that might interfere with participation. Four hundred and ninety two patients were determined eligible for the study by their treating clinicians based on clinical evaluation and judgment of inclusion and exclusion criteria. Of potentially eligible patients, 346 patients (70%) consented and participated in initial assessment. Reasons for non-participation were primarily patients unwilling or unable to be reached. A few patients were excluded from the study prior to completing initial assessment due to inadequate English or language difficulties impeding their understanding the consent and/or study questions. At initial assessment, patients were administered questionnaires assessing demographics and trait characteristics, psychiatric symptomology, and STB. Patient participants were contacted one month after initial assessment for follow-up assessment of STB. Of the patient participants who completed the initial assessment, 276 (78.6%) completed follow-up assessment. The Icahn School of Medicine at Mount Sinai, Mount Sinai Beth Israel, and Mount Sinai St. Luke’s—Roosevelt Institutional Review Boards approved this study.

### Measures

#### Clinician Emotional Response

The Therapist Response Questionnaire-Suicide Form [TRQ-SF (25)] is a 10-item, Likert-type scale designed to capture clinicians’ emotional responses to acutely suicidal patients. TRQ-SF individual item scores range from 0 (not at all) to 4 (extremely). Two factors were identified in previous study: affiliation (items 1, 2, 4, 8, and 10) and distress (items 3, 5, and 7) and a third *a priori* factor of hopefulness (items 6 and 9) (25). A total score of all TRQ items, with positively worded items reverse scored reflected negative emotional responses. Possible ranges for TRQ affiliation, distress, hope and total scores were 0–20, 0–12, 0–8, and 0–40, respectively.

#### STB Criteria

The Columbia Suicide Severity Rating Scale [CSSRS (30)] is a semi-structured interview of current and past STBs. The CSSRS outlines five progressively more severe levels of ideation, and three types of suicidal behaviors (SB) are defined: actual, interrupted and aborted suicide attempts. A composite variable of STBs on a 0–9 point scale was used: a score of 0–5 based on their peak level of suicidal ideation (SI) in the past month. Preparations for suicide without attempt received a score of 6. An aborted, interrupted, or actual suicide attempt in the past month received a score of 7–9, respectively (25, 31).

The Beck Scale for Suicide Ideation [BSS (32, 33)], a 21-item self-report measure of active and passive suicidal desires, was used. Because items 6–21 are completed only if items 4 and 5 are rated >0, we used BSS part 1 scores (items 1–5) in lieu of the total scale score. The possible range is 0–10. The BSS part 1 demonstrated good internal consistency in our sample (α = 0.83).

The Clinician Prediction Scale [CPS (34, 35)] was used to measure clinician judgment of patient suicide risk. This single-item scale asks clinicians to rate the likelihood of their patients making a suicide attempt in the next 6 months if untreated, on a scale ranging from 0 (no likelihood) to 10 (very high likelihood).

#### Secondary Criteria

The Beck Depression Inventory [BDI (36, 37)] is a widely used 21-item self-report measure of severity of depressive symptoms. Total score ranges for severity have been recommended: 14–19 (mild), 20–28 (moderate), and 29–63 (severe). The BDI demonstrated excellent internal consistency in our sample (α = 0.91).

The Brief Symptom Inventory [BSI (38)] is a 53-item self-report measure of psychiatric symptoms which provides a reliable measure of global severity of psychopathology. Patient participants completed the BSI at initial assessment only. The BSI demonstrated excellent internal consistency in our sample (α = 0.97).

#### Convergent and Discriminant Validity Measures (Clinician Report)

The State-Trait Anxiety Inventory (39, 40) is a 40-item self-report assessment of state and trait levels of anxiety. Both subscales demonstrated good internal consistency (trait α = 0.84, state α = 0.95).

The Spielberger State-Trait Anger Expression Inventory [STAXI (41)] is a 44-item self-report assessment of state and trait anger and anger expression. Internal reliability of both the trait (α = 0.73) and state (α = 0.85) subscales was acceptable in our sample.

The Big Five Inventory (42) is a 44-item self-report assessment of the “Big Five” personality dimensions: Extraversion, Agreeableness, Conscientiousness, Neuroticism, and Openness. The five subscales demonstrated acceptable internal reliability in our study (α ranged from 0.77 to 0.84).

The WAI (28, 43) is a measure of patient-therapist alliance comprising three components: bond, and agreement on goals and tasks. For the current analyses we excluded the two items that were included in the TRQ-SF. Internal reliability (α = 0.93) was excellent in our sample.

### Statistical Analyses

#### Test of Assumptions

We conducted preliminary exploration of our data to identify any constraints in our sample that would impact our choice of statistical test. TRQ-SF responses were both univariate and multivariate non-normal, contraindicating the use of parametric techniques, thus all analyses were conducted with a parallel non-parametric test. Substantial TRQ-SF intraclass correlations (ICCs) suggested that there was appreciable variation in responses attributable to individual clinician differences (see “Results” for detail). While this would indicate the use of multilevel modeling given the clustering of patient TRQ-SF ratings within clinicians, this approach would not be appropriate due to Type I error inflation given the relatively small sample of clinicians and large proportion of clinicians referring only a few patients (44, 45). We, therefore, report only single-level model results. It is of note that the multilevel results did not substantively differ from the single-level results presented (available from the authors by request).

#### Analysis of Factor Structure

A Confirmatory Factor Analysis (CFA) was conducted to test our hypothesized factor structure. We used diagonally weighted least squares estimation, which is most appropriate for Likert scale and multivariate non-normal responses (46, 47). Absolute model fit was evaluated with the chi-squared statistic, which indicates the degree of agreement between the observed and expected covariance matrices and thus a non-significant test supports good model fit. Because of the sensitivity of the chi-squared test to sample and model size, additional indices of relative fit were evaluated, including the Comparative Fit Index (CFI) the Tucker-Lewis Index [TLI (48)], and the Root Mean Square Error of Approximation [RMSEA (49)]. Based on conventional standards, good fit is indicated by CFI and TLI values greater than 0.95 and an RMSEA value below 0.07 with a 90% confidence interval lower bound less than 0.05 and upper bound less than 0.10 (48, 50, 51). The CFA was conducted in the *lavaan* package of *R* statistical software (52).

#### Convergent, Discriminant, and Criterion Validity

Spearman’s *rho* was used to investigate the 0-order rank correlations between the TRQ-SF subscales and total score and the convergent- and discriminant-related variables. A hierarchical linear regression analysis, with trait scales entered into the first step and state scales entered into the second step, was employed to evaluate whether clinicians’ trait-variables predicted TRQ-SF scores controlling for clinician state-variables. Because TRQ-SF scores substantially deviated from the normal distribution, we used a computed *log* function of TRQ-SF as the dependent variable. To examine TRQ-SF scores, cross-sectional, and prospective associations with patient suicide outcome measures, and concurrent clinician assessments of patient suicide risk and psychopathological symptom severity, we performed another series of 0-order correlations by Spearman’s *rho*. Convergent, discriminant, and criterion validity analyses were performed with SPSS version 24.0.

## Results

### Participant Characteristics

Patient and clinician characteristics are described in Table 1.

**Table 1:** Patient demographics and descriptive measures.

Demographics*n*(%) or mean (SD), as appropriate	Whole sample (*N* = 346)	Lost to follow-up (*n* = 79)	Followed-up (*n* = 267)	*p*-Value
Gender (female)	225 (65.0)	54(68.4)	171 (64.0)	0.74
Race				0.26
Asian	24 (6.9)	4 (5.1)	20 (7.5)	
Black	97 (28.0)	28 (35.4)	69 (25.8)	
White	124 (35.8)	21 (26.6)	103 (38.6)	
Other	93 (26.9)	24 (30.4)	69 (25.8)	
Age	39.29 (14.1)	34.09 (12.7)	40.78 (14.1)	<0.01
Annual household income				0.72
<$20,000	195 (56.4)	45 (57.0)	150 (56.2)	
$20–39,000	69 (19.9)	14 (17.7)	55 (20.6)	
$40–59,000	30 (8.7)	7 (8.9)	23 (8.6)	
$60–79,000	17 (4.9)	3 (3.8)	14 (5.2)	
$80–99,000	12 (3.5)	1 (1.3)	11 (4.1)	
>$100,000	15 (4.3)	5 (6.3)	10 (3.7)	
Years of education	14.3 (3.3)	13.6 (3.2)	14.5 (3.3)	0.04
**Patient baseline clinical characteristics**				
Primary diagnosis				0.08
Depressive disorder	156 (45.1)	29 (36.7)	127 (47.6)	
Anxiety disorder	34 (9.8)	5 (6.3)	29 (10.9)	
Bipolar disorder	44 (12.7)	8 (10.1)	36 (13.5)	
Psychotic disorder	26 (7.5)	5 (6.3)	21 (7.9)	
Trauma disorder	52 (15.0)	18 (22.8)	34 (12.7)	
Other	15 (4.3)	4 (5.5)	11 (4.1)	

**Descriptives*n*(%) or mean (SD), as appropriate. Observed response ranges reported**				

**Patient Baseline**				
Lifetime SA^a^	144 (41.6)	27 (34.2)	117 (43.8)	0.13
SI^b^ (range 0–9)	1.73 (2.3)	1.88 (2.5)	1.69 (2.2)	0.53
Depression (range 0–54)	22.44 (12.2)	23.25 (12.2)	22.21 (12.2)	0.52
Global Severity (range 53–424)	135.68 (47.7)	142.04 (42.4)	134.09 (47.7)	0.24
Lifetime STB^c^ (range 0–9)	5.09 (3.5)	4.58 (3.5)	5.25 (3.5)	0.12
Past month STB^c^ (range 0–9)	1.61 (2.2)	1.76 (2.6)	1.57 (2.1)	0.50
**Patient follow-up**				
SI^b^ (range 0–8)			1.29 (1.9)	
Depression (range 0–50)			19.09 (11.6)	
STB^c^ since baseline (range 0–9)			2.00 (1.7)	
**Clinician patient-level ratings**				
TRQ-SF^d^ Total (range 0–33)	9.12 (5.2)	9.36 (6.0)	9.05 (5.0)	0.65
Affiliation (range 2–20)	13.84 (3.2)	13.65 (5.5)	13.90 (3.1)	0.55
Distress (range 0–9)	1.08 (1.7)	0.95 (1.8)	1.11 (1.6)	0.45
Hope (range 2–8)	6.14 (1.1)	6.05 (1.2)	6.17 (1.0)	0.40
Working alliance (range 13–84)	58.32 (14.2)	58.94 (15.3)	58.04 (13.9)	0.62
State Anxiety (range 20–64)	30.86 (9.5)	29.46 (10.9)	31.40 (9.0)	0.22
State Anger (range 15–35)	16.00 (2.4)	15.78 (2.2)	16.04 (2.4)	0.42
Assessment of Risk (range 0–8)	1.79 (1.7)	2.37 (2.2)	1.54 (1.4)	<0.01
**Clinician traits**				
Trait anxiety (range 27–55)	39.0 (7.0)			
Trait anger (range 10–26)	16.73 (3.6)			
Extraversion (range 14–38)	25.59 (6.3)			
Agreeableness (range 24–45)	35.51 (5.9)			
Conscientiousness (range 20–44)	34.85 (5.4)			
Neuroticism (range 9–31)	21.76 (5.7)			
Openness (range 25–57)	38.80 (6.7)			

*^a^Suicide Attempt assessed with the Columbia Suicide Severity Rating Scale (CSSRS)*.

*^b^Suicidal Ideation (SI) assessed with the Beck Scale for Suicide Ideation*.

*^c^Suicidal Thoughts and Behaviors assessed with the CSSRS*.

*^d^Therapist Response Questionnaire-Suicide form*.

#### Patients

The baseline sample included 346 subjects. Of those, 267 (77.2%) completed follow-up assessment 1 month following the initial assessment. Participants reached for follow-up were more likely to be older (mean difference 6.7 years, *p* < 0.001) and have, on average, 1 more year of education (*p* = 0.04) than those lost to follow-up. There were no other differences in sociodemographic characteristics. Descriptive statistics for all included scales are reported in Table 1. More than 40% of participants had made a suicide attempt in their lifetime. The average level of peak lifetime STB was active SI with plan and intent, while the average past-month level at intake was between passive wishes to be dead and active SI without methods, plan, or intent. On average, our participants fell into the moderate depressive severity range at both intake and follow-up. Participants lost to follow-up had been judged by clinicians to be at slightly higher risk for suicide (*p* = 0.004). There were no other differences in clinical characteristics between the groups, including measures of global severity, depression, and SI, or in TRQ-SF responses, however.

#### Clinicians

The clinician sample consisted of 48 mental health trainees and professionals of whom 22 (45.8%) were women (4 participants were missing gender information), 20 (41.7%) were white, and 28 (58.3%) were born in the US. The participating clinicians were 83.3% psychiatrists (including 38 third-year psychiatry residents and two attending psychiatrists), 4.2% attending psychologists, and 4.2% social workers. The primary theoretical orientations reported by clinicians were 22.7% psychodynamic, 20.5% cognitive/behavioral, 13.6% integrative, 6.8% humanistic/supportive, and 4.5% interpersonal. The average reported length of clinical experience as a clinician was 4.05 years (SD = 4.79, range = 0–22).

Scores on the TRQ-SF were, in part, accounted for by clinician-level differences, as indicated by the ICCs. ICCs ranged from 0.16 (item 5: “I felt guilty about my feelings toward him/her”) to 0.40 (item 6: “I thought life really might not be worth living for him/her”), indicating that between 16 and 40% of the total variance in ratings of each TRQ-SF item can be attributed to individual clinician differences (vs. individual patient differences).

### Confirmatory Factor Analysis

A CFA was conducted to evaluate the proposed three-factor model (see Table 2) with a sample of 346 complete TRQ-SF ratings and the first indicator loading (i.e., item 1 in the affiliation factor, item 3 in the distress factor and item 9 in the hope factor) fixed to 1. All three of the approximate fit indexes (CFI, TLI, and RMSEA) exceeded the recommend threshold values for good fit, suggesting that the three-factor model fit the data well. The test of exact fit was significant, therefore not supporting exact model fit, χ^2^(32) = 54.06 (*p* = 0.009), which is unsurprising as the chi-square test is very sensitive to sample size.

**Table 2:** Goodness-of-fit indices for the TRQ-SF (*N* = 346).

Model	χ2	df	*p*-Value	CFI^a^	TLI^b^	RMSEA^c^ (95% CI^d^)
1 Factor^e^	63.65	35	0.002	0.979	0.973	0.049 (0.029, 0.067)
3 Factor^f^	54.06	32	0.009	0.984	0.977	0.045 (0.023, 0.065)
1 vs. 3^g^	9.59	3	0.022			

*^a^Comparative Fit Index [good fit indicated by Comparative Fit Index (CFI) > 0.95]*.

*^b^Tucker-Lewis Index (good fit indicated by TLI > 0.95)*.

*^c^Root Mean Square Error of Approximation (good fit indicated by RMSEA < 0.05)*.

*^d^Confidence interval of RMSEA (good fit indicated by lower bound CI < 0.05, upper bound CI < 0.10)*.

*^e^One-factor model of clinicians’ emotional responses with all 10 items of the Therapist Response Questionnaire-Suicide form (TRQ-SF) serving as indicators*.

*^f^Three-factor model with correlated factors: affiliation (Items 1, 2, 4, 8, and 10), distress (Items 3, 5, and 7), and hope (Items 6 and 9)*.

*^g^Comparison of the one- and three-factor models using the chi-square difference test*.

**Figure 1:**
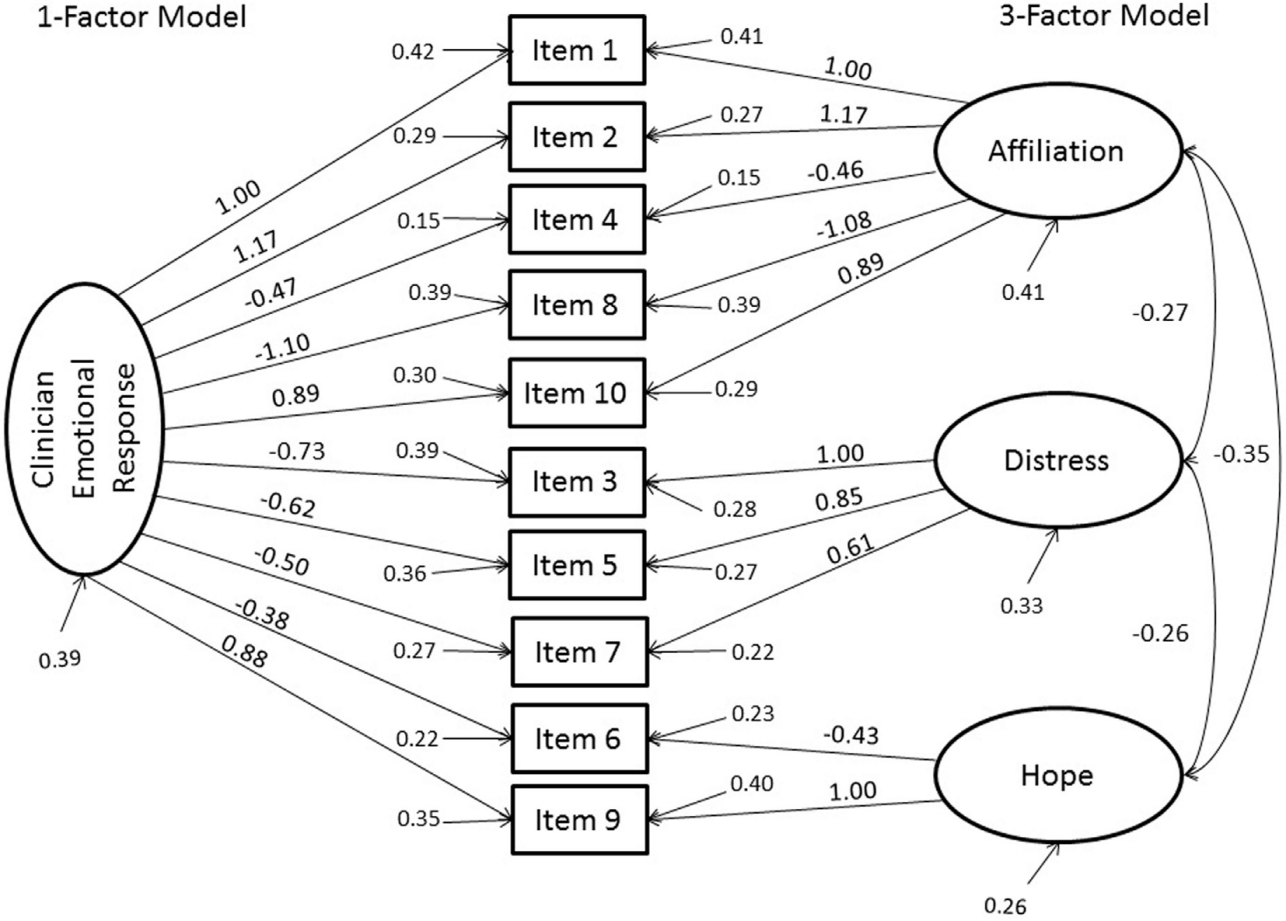
Results of the confirmatory factor analyses of the one and three-factor models of Clinicians’ Emotional Responses to their patients using items from the Therapist Response Questionnaire-Suicide Form (TRQ-SF). Factor loadings, variances, and covariances and indicator variances are reported. All factor loadings were significant in both models, as were the factor covariances in the three-factor model (*p* < 0.001).

As the three subscales were substantially correlated (*|r*| = 0.55 −0.68; *p* < 0.001), suggestive of a single general factor with three correlated subfactors, we conducted an additional CFA to test the model fit of the TRQ-SF as one-factor. The one-factor model also exceeded the recommend threshold values for good fit by approximate fit indexes (CFI, TLI, and RMSEA) but the test of exact fit was significant. A chi-square difference test revealed that the three-factor model provided slightly yet significantly better fit than the one-factor model, [χ^2^(3) = 9.59, *p* = 0.022], supporting the common factor with three subfactor interpretation. Factor loadings are reported in Figure 1.

We computed Cronbach’s alpha for the three subscales in SPSS version 24. Alpha was adequate for the affiliation (0.83) and distress (0.71) subscales. However, alpha was low for the hope subscale (0.37), likely as a result of only two items being included in this subscale (53). For the total score, we computed both Cronbach’s alpha and Revelle’s beta (54) in the *psych* package for R (55) to evaluate internal consistency of the full scale. Beta is a lower bound estimate of reliability, whereas Cronbach’s alpha is a measure of the average split-half reliability; therefore, minimal discrepancy between alpha and beta, specifically a discrepancy less than 15–20 points (56), suggests factorial homogeneity. This analysis produced an alpha value of 0.88 and a beta value of 0.75, indicating good internal consistency and scale unidimensionality. Taken together, these results suggest that there are three distinct, but highly correlated factors that combine into a meaningful general factor. We, therefore, found it appropriate to explore validity of both the TRQ-SF total score and all three subscales.

Examination of the descriptive statistics indicated there was somewhat of a floor-effect in terms of total scale and distress subscale ratings, with average responses falling in the “not at all” to “a little” range of the Likert scale. Average responses to the affiliation and hope subscale were more moderate, falling in the “somewhat” to “quite a bit” range.

### Convergent and Discriminant Validity

To further examine the convergent and discriminant validity of the TRQ-SF general scale and subscales, we conducted 0-order correlation analyses. As shown in Table 3, the TRQ-SF total scores were positively related with clinicians’ *state* anxiety and anger, and negatively correlated with clinician report of the therapeutic alliance. The magnitude of these associations was moderate to strong with high levels of significance (i.e., *p* ≤ 0.001). TRQ-SF subscales demonstrated the same magnitude and significance of associations in the expected directions: affiliation and hope were negatively associated with clinician *state* anxiety and anger, and positively associated with therapeutic alliance scores. The results for the clinician trait variables showed that (a) the *trait* anxiety and anger were significantly negatively associated with TRQ-SF total scores and (b) TRQ-SF total scores were significantly negatively associated with clinician agreeableness and positively associated with clinician neuroticism and openness. The distress subscale showed comparable results as the total score, except for significant negative association with clinician extraversion, and non-significant association with openness. The affiliation and hope subscales demonstrated similar significance in the opposite direction of associations compared with the TRQ-total, except for a non-significant association between affiliation and clinician trait anxiety, and between hope and clinician trait anger. Overall, the associations between clinicians’ traits and TRQ-SF were modest. Consistent with the pattern of these results, we sought to examine the contribution of clinician personality traits to TRQ-SF scores controlling for state-specific effects. We, therefore, conducted a hierarchical linear regression analysis predicting TRQ-SF total score. The results, presented in Table 4, show that among clinician traits included in the first step, clinician extroversion, and agreeableness were negatively associated with negative emotional responses to patients, while clinician openness was positively associated. On the second step, including state-specific measures, only state anxiety *(beta* = 0.214, *p* = 0.001) and clinician report of therapeutic alliance (*beta* = −0.621, *p* < 0.001) were independently associated with clinicians’ overall negative emotional responses as measured by the TRQ-SF total score.

**Table 3:** Convergent/discriminant validity: 0-order correlations between clinicians’ variables and TRQ-SF.

	Total score	Affiliation	Distress	Hope
State anxiety	0.51**	−0.42**	0.55**	−35**
Trait anxiety	0.17**	−0.10	0.23**	−0.18**
State anger	0.60**	−0.53**	0.55**	−0.35**
Trait anger	0.25**	−0.29**	0.14*	−0.10
Working alliance	−0.77**	0.74**	−0.50**	0.67**
Extroversion	−0.05	−0.04	−0.23**	0.04
Agreeableness	−0.24**	0.27**	−0.13*	0.13*
Conscientiousness	0.01	−0.04	−0.09	−0.01
Neuroticism	0.25**	−0.22**	0.20**	−0.24**
Openness	0.22**	−0.25**	0.05	−0.26**

*p < 0.05.

**p < 0.01.

**Table 4:** TRQ-SF total convergent/discriminant associations with clinician traits and patient-specific states.

Predictor variable	*B*	SE *B*	β	*t*	Sig.
**Model 1: clinician trait measures^a^**					
Trait anxiety	−0.001	0.009	−0.014	−0.144	0.885
Trait anger	0.004	0.015	0.025	0.264	0.792
Extroversion	−0.022	0.007	−0.274	−3.316	0.001
Agreeableness	−0.023	0.009	−0.232	−2.477	0.014
Conscientiousness	−0.013	0.008	−0.123	−1.723	0.086
Neuroticism	0.016	0.011	0.141	1.387	0.167
Openness	0.019	0.006	0.262	3.300	0.001
**Model 2: clinician state and trait measures^b^**					
Trait anxiety	0.002	0.006	0.021	0.350	0.727
Trait anger	0.007	0.010	0.043	0.708	0.479
Extroversion	−0.001	0.004	−0.009	−0.166	0.868
Agreeableness	−0.004	0.006	−0.041	−0.684	0.495
Conscientiousness	0.002	0.005	0.016	0.347	0.729
Neuroticism	−0.012	0.007	−0.108	−1.668	0.097
Openness	0.006	0.004	0.087	1.711	0.088
State anxiety	0.013	0.004	0.214	3.403	0.001
State anger	0.017	0.010	0.086	1.640	0.102
Working alliance	−0.399	0.032	−0.621	−12.483	0.000

*^a^R^2^ = 0.379, p < 0.001*.

*^b^R^2^ = 0.820, p < 0.001, ΔR^2^ = 0.441, p < 0.001*.

### Criterion Validity

#### Concurrent and Prospective Criterion Validity

The TRQ-SF demonstrated statistically significant 0-order correlations with each of the criterion-related variables, except for the global psychopathological symptom severity. The associations ranged in value from.12 (*p* < *0.05*) to 0.33 (*p* < *0.01*) (see Table 5). Results, therefore, supported the concurrent and prospective criterion validity for the primary outcomes and demonstrate the relative specificity of the TRQ-SF response to clinician-perceived and patient-reported suicide risk.

**Table 5:** Criterion validity of the TRQ-SF total, three factors and individual items: 0-order correlations between patient variables and TRQ-SF.

	Concurrent (*N* = 346)						Prospective (*N* = 267)		
	Assessment of risk	STB lifetime	STB recent	SI	Depression	Global severity	STB	SI	Depression
TRQ-SF total	0.33**	0.21**	0.16**	0.21**	0.20**	0.10	0.12*	0.22**	0.25**
Affiliation	−0.29**	−0.20**	−0.16**	−0.20**	−0.20**	−0.10	−0.07	−0.16**	−0.20**
Distress	0.33**	0.12*	0.10	0.10	0.13*	0.07	0.06	0.18**	0.22**
Hope	−0.22**	−0.12*	−0.08	−0.09	−0.13*	−0.07	−0.13*	−0.16*	−0.19**
**Individual items**									
1-Feel good	−0.20**	−0.14**	−0.18**	−0.13*	−0.13*	−0.05	−0.10	−0.09	−0.12*
2-Liking	−0.24**	−0.14*	−0.11*	−0.14**	−0.20**	−0.09	−0.04	−0.10	−0.20**
3-In a bind	0.22**	0.08	0.09	0.07	0.08	0.01	0.05	0.13*	0.19**
4-Devalued	0.30**	−0.01	0.05	0.03	0.10	0.02	0.01	0.07	0.09
5-Guilty	0.29**	0.15**	0.06	0.10	0.12*	0.09	0.07	0.11	0.13
6-Not worth living	0.22**	0.12*	0.12*	0.07	0.05	−0.00	0.13*	0.10	0.04
7-Chills	0.25**	0.04	0.10	0.13*	0.15**	0.08	0.08	0.22**	0.18**
8-Forced self	0.23**	0.16**	0.04	0.10	0.17**	0.09	0.00	0.18**	0.18**
9-Confident	−0.20**	−0.08	−0.04	−0.07	−0.14*	−0.08	−0.09	−0.15*	−0.20**
10-Trust	−0.22**	−0.21**	−0.17**	−0.19**	−0.12*	−0.09	−0.08	−0.11	−0.11

*p < 0.05.

**p < 0.01.

*STB, suicidal thoughts and behaviors; SI, suicidal ideation*.

Finally, we conducted exploratory analyses for the concurrent and predictive validity of each individual TRQ-SF item (see Table 5). Overall, results indicate significant cross-sectional associations between all the scale items and clinician judgment of patient suicide risk. None of the items associated with patient global symptom severity. Eight out of ten single items (except for items 3 and 4) were significantly associated with at least one of the concurrent suicidal severity measures. Items 3, 7, 8, and 9 were associated with SI at 1-month follow-up. Item 6 (“I thought life really might not be worth living for him/her”) was exclusively associated with STB both at intake and at 1-month follow-up, while not associated with depression and global symptom severity, showing suicide-specific reaction.

## Discussion

This study replicated, validated and generalized the performance of the novel TRQ-SF for assessing a distinct suicide-related countertransference in a general outpatient clinic setting—a clinical sample with low and moderate short-term suicide risk. Adding to our previous reports on an acute inpatient sample (25, 29), these emotional responses are found to be related to patient risk for suicide as judged during the clinical assessment as well as predictive of short-term risk for SI and behavior. The current study results support the psychometric performance, construct and predictive validity of the TRQ-SF. Further, as they are generally consistent with our previous results in a high acuity patient population, our findings broadly support generalizability of the TRQ-SF across different clinical settings. Thus, our findings suggest that the TRQ-SF may be of diagnostic and therapeutic use.

### Factor Structure and Reliability

First, we found good fit for the hypothesized 3-factor model including affiliation, distress, and hope, thus supporting our predictions based on our previous study in a high suicide-risk inpatient sample (25). The results also supported the performance of the TRQ-SF as a general one-factor scale in assessing overall negative emotional responses toward the patient. While the three-factor model has slightly better fit compared to the one-factor model, the high inter-correlations between the three subscales and the high internal consistency indices that were found for the full scale support the meaningfulness and reliability of a scale total score. These results are in line with our previous studies using the TRQ-SF total score (9, 29). Findings, therefore, suggest that the TRQ-SF may be used as an overall scale to assess clinicians’ emotional responses toward suicidal patients, as well as to assess specific dimensions of these emotional responses as indicated by the affiliation, distress, and hope subscales. While the brevity of the scale is one of its advantages for research and clinical use, given the small number of items, a larger item pool may be needed to reliably probe correlated subfactors.

### Convergent and Discriminant Validity

Convergent and discriminant validity analyses primarily supported our predictions. Specifically, we found that the TRQ-SF associated significantly with related measures of general negative emotional states, i.e., anger and anxiety, and negative assessment of the therapeutic alliance. Patterns of correlation did not differ substantially between hypothesized subfactors, again supporting a generally unidimensional suicide-related countertransference, although some variation was observed, particularly in relation to clinician personality traits.

The considerable magnitude of the ICCs indicates that there was significant variation in responses attributable to individual clinician differences. Indeed, clinicians’ emotional responses to patients as measured by the TRQ-SF were related to clinicians’ individual tendencies to experience such emotions, as evidenced by the significant 0-order associations with clinician trait anxiety, anger, agreeableness, neuroticism, and openness. However, when accounting for state anger, anxiety, and therapeutic alliance as reported immediately following the clinical interaction with the patient, the contribution of any of the clinician’s traits was no longer significant. This suggests that the effects of personality on clinician TRQ-SF responses are mediated by the specific emotional states to which they are prone. For example, we suggest that the association between trait anxiety and the TRQ-SF may be explained by the correlation between state anxiety and the TRQ-SF. The findings are consistent with a relational understanding of countertransference as clinicians’ emotional responses cocreated in interaction with the patient; “The patient draws the therapist into playing a role that reflects the patient’s internal world, but the specific dimensions of that role are colored by the therapist’s own personality.” [(6) p. 981]. It is of note that TRQ-SF scores were most strongly associated with clinician-reported state anxiety and lower therapeutic alliance. Both are robustly documented as prevalent in clinical interactions with suicidal individuals (14) as well as with poor treatment outcomes (2, 4, 7, 57).

### Criterion Validity: Diagnostic and Prognostic Usefulness of the TRQ-SF

We found modestly sized but highly significant correlations of TRQ-SF total and its subscale scores with patient suicide outcomes. These correlations were stronger than associations with global severity of patient psychopathology, supporting a specifically suicide-related countertransference. Although similar associations with severity of depression were found, depression severity independent of clinical diagnosis is itself a robust predictor of SI and suicide (58). TRQ-SF scores were associated with suicide risk indices both as reported by the patient (i.e., STBs) and as assessed by the clinician (i.e., CPS). These findings support the potential utility of this scale for diagnosis and therapeutic interventions. This is in line with the body of psychological literature suggesting that evaluating therapists’ countertransference is beneficial to understanding patients’ intra-psychic and interpersonal dynamics (59–62). These results are also consistent with studies of countertransference measures, for example the Therapist Response Questionnaire, showing that countertransference reactions measured by clinician’s self-report questionnaire were able to systematically capture patient personality and severe psychopathology regardless of therapeutic orientation (3, 63). Moreover, the prospective associations of the TRQ-SF with patient STB at 1-month following the initial intake demonstrate its potential clinical value in predicting short-term STB. These results support and extend our previous studies showing post-discharge SB among high-risk inpatients were predicted by clinicians’ emotional responses as measured by the TRQ-SF (25, 29). This study, therefore, validates and generalizes our previous findings to a general psychiatric outpatient population with lower suicide-related acuity level. Hence, even in a sample with low base rate of SI and behaviors, clinicians’ emotional responses measured by the TRQ-SF predict near-term STBs.

Exploratory examination of the criterion validity of individual TRQ-SF items suggests that items consistent with a projective identification process had very modest but significant prospective criterion validity, including feelings of entrapment (“My hands were tied or I was put in an impossible bind”), hopelessness (“I thought life really might not be worth living for him/her”), and helplessness (“I felt confident in my ability to help him/her”, reversed) as well as unique affective-arousal (“patient gave me chills”). Items indicative of interpersonal rejection/difficulties (e.g., “I had to force myself to connect”) also demonstrated modest concurrent criterion validity. Moreover, the item “I thought life really might not be worth living for him/her” showed unique suicide-specific associations and, therefore, future development may aim to better probe this emotional domain. Overall, the pattern of emotional reactions indicated by the individual items may relate to specific affective characteristics of the suicidal crisis as described in previous research (25, 64, 65). Joiner and Stanley (66) proposed that these suicidal affective states may activate an emotional contagion through non-verbal and perhaps unconscious mechanisms (66). Therefore, these intense reactions may alert a clinician to patient suicide risk independent of overtly evident risk factors such as declared suicidal intent, reported access to means, and so on. At the same time, negative emotional responses may result in less empathic communication, avoidance, and unwitting rejection of the patient (67–69) which are liable to damage the therapeutic alliance (70). Thus, further research is needed to elucidate the mechanisms by which clinicians’ negative emotional responses are associated with patients’ subsequent suicidal outcomes.

### Implications

Our results are broadly consistent with the literature that suggests clinicians have negative emotional responses to patients presenting as at risk for suicide (14–18, 25, 67), and deepen this literature by characterizing specific responses that associate prospectively both individually and in aggregate with suicide-related outcomes and clinician judgment of suicide risk. Further, our results extend the growing body of literature that supports the potential diagnostic specificity of emotional responses (5, 6, 34, 44–48, 50, 53–56).

Mental health professional who routinely encounters patients at risk for suicide may benefit from recognizing and evaluating their emotional responses to their patients. Moreover, making meaning of emotional responses to patients is critical to managing them in a way that benefits the therapeutic relationship (59–62). Integrating explicit knowledge with the information coded in emotional cues to enhance understanding and care for suicidal individuals in training curricula and continuing education programs may facilitate assessment, intervention and ultimately reduce the risk of suicide. Training interventions for clinicians may focus on emotional self-awareness, such as mindfulness training for mental health professionals and trainees (71); relational therapy interventions that focus on the therapeutic alliance and managing counter-transference responses (72, 73) as well as novel training methods designed for improving empathic communication with psychiatric patients (74). In addition, existing training programs for suicide prevention (75) would benefit from including a component targeting emotional responses to suicidal individuals. Intervention emphasizing emotional self-awareness and the effect of clinicians’ emotions on the clinical interaction may help clinicians to better recognize and assess the emotional response to their patients as well as to make use of this emotional awareness in a way that would benefit both evaluation and treatment.

### Strengths and Limitations

This study has a number of strengths and limitations. Among the strengths of the study are the use of multiple validated measures of patient SI, assessment of both patient and clinician characteristics, moderately large sample size, and high rates of follow-up. Several limitations should also be noted. First, our analyses suggested that individual clinician differences are a considerable element of the TRQ-SF reports; however, multilevel modeling accounting for clinician individual differences was limited by the fact that many clinicians reported on only few patients, while a few clinicians reported on many patients. Further study is needed to evaluate the nature and extent of individual clinicians’ effects. A second limitation of the study is the low rate of suicidal behavior at follow-up such that prediction of suicide attempts was not powered sufficiently to be tested. Third, while clinician responses were collected anonymously, social desirability bias cannot be ruled out. Finally, while we examined patient psychopathology symptoms, patient personality psychopathology was not broadly examined and accounted for in interpreting the results.

### Conclusion

The primary implication of this study is that mental health professionals’ emotional responses to their patients are indicative of severity of patients’ STBs as well as predictive of the short-term development of these thoughts and behaviors. Our results support the potential utility of the TRQ-SF as a reliable, easily used and scored measure of clinicians’ suicide-related countertransference. The brevity of this self-assessment may make it particularly useful in the clinical setting and for longitudinal clinical research as it may be comfortably administered on a repeated basis. Indeed, further study is warranted to understand how changes in countertransference over time such as might be assessed with the TRQ-SF in a series of patient encounters relate to changes in patient motivation for or liability to suicide.

## Ethics Statement

The study protocol was approved by the The Icahn School of Medicine at Mount Sinai, Mount Sinai Beth Israel, and Mount Sinai St. Luke’s—Roosevelt Institutional Review Boards. All subjects gave written informed consent in accordance with the Declaration of Helsinki.

## Author Contributions

SB and ZY conducted and contributed to the design of the experiment, designed the data analyses, analyzed the data, and wrote the paper. MH conducted the experiment and designed the data analyses, analyzed the data, and wrote the paper. BG designed the data analyses, analyzed the data, and contributed to writing the paper. RA, AF, and AA contributed to writing the paper. PR conducted the experiment. IG conducted and designed the experiment and contributed to writing the paper.

## Conflict of Interest Statement

The authors declare that the research was conducted in the absence of any commercial or financial relationships that could be construed as a potential conflict of interest.
